# Heightened anti-inflammatory and antioxidant effects of bone marrow stem cell-conditioned media with mono and doped TiO_2_ nanoparticles in Carrageenan-induced inflammation

**DOI:** 10.1007/s10787-025-02020-5

**Published:** 2025-12-12

**Authors:** Mohamed S. Kishta, Ahmed M Youssef, Mohamed I. El-Khonezy, Soheir E. Kotob, Nayera E. Hassan, Ahmed A. Abd-Rabou

**Affiliations:** 1 Hormones Department, Medical Research and Clinical Studies Institute, National Research Centre, Dokki, Cairo 12622 Egypt; 2 Stem Cell Lab, Center of Excellence for Advanced Sciences, National Research Centre, Dokki, Cairo 12622 Egypt; 3 Inorganic Chemistry Department, Inorganic Chemical Industries and Mineral ResourcesResearch Institute, National Research Centre, Dokki, Cairo 12622 Egypt; 4 Molecular Biology Department, Biotechnology Research Institute, National Research Centre, Dokki, Cairo 12622 Egypt; 5 Medical Research Division, Department of Biological Anthropology, National Research Center, Cairo, Egypt

**Keywords:** TiO₂ nanoparticles, BM-MSCs, Inflammation, And carrageenan

## Abstract

Inflammation and oxidative stress are key mediators of tissue damage in numerous pathological conditions. This study investigated the anti-inflammatory and antioxidant effects of titanium dioxide nanoparticles (TiO₂ NPs), mono-doped TiO₂ (Cu), and dual-doped TiO₂ (Cu/Zn) in a carrageenan-induced paw edema model. TiO₂ nanoparticles were synthesized and characterized by XRD, SEM, TEM, DLS, and EDX, confirming an anatase crystalline phase, spherical morphology, and uniform size distribution with successful dopant incorporation. Bone marrow mesenchymal stem cells (BM-MSCs) were isolated and characterized by flow cytometry (CD90⁺, CD73⁺, CD34⁻, CD45⁻) and trilineage differentiation, and their conditioned media (CM) were used for therapeutic application. A pre-study screening using three doses (10, 20, and 50 mg/kg) demonstrated a dose-dependent reduction in paw edema, with the 50 mg/kg dose showing the highest inhibition, comparable to indomethacin. The main experiment comprised six groups: control, carrageenan, indomethacin, TiO₂ CM, Cu–TiO₂ CM, and Cu/Zn–TiO₂ CM. Carrageenan administration elevated TNF-α, IL-6, IL-4, COX-2, and 5-LOP while reducing IL-10, alongside increased oxidative stress markers (MDA and NO) and decreased antioxidant defenses (CAT, SOD, GPx, and TAC). Treatment with TiO₂ formulations markedly reversed these effects in a dose-dependent manner. Dual-doped TiO₂ combined with BM-MSC-CM produced the greatest improvement, normalizing cytokine profiles, reducing lipid peroxidation and nitric oxide levels, and restoring antioxidant enzyme activities. Histopathological assessment confirmed these findings, showing nearly normal dermal architecture with minimal inflammatory infiltration. The results highlight the synergistic therapeutic potential of doped TiO₂ nanoparticles and BM-MSC-CM in mitigating inflammation and oxidative stress.

## Introduction

Inflammation is one of the most common processes that occurs in the body. Inflammation occurs as a normal part of the tissue healing process, but exaggerated inflammation or chronic inflammation that goes on for long periods is harmful and is involved in numerous diseases such as rheumatoid arthritis, diabetes, and atherosclerosis. Inflammation occurs through several intertwined pathways that include the secretion of numerous pro-inflammatory mediators such as cytokines, leukotrienes, and prostaglandins. Measurement of some of these molecules, including IL4, IL6, IL10, TNF-a, Cyclooxygenase 2 (COX-2), and 5-Lipoxygenase (5-LOP) could serve as a biomarker of inflammatory status (Patil et al. 2019).

Mesenchymal stem cells (MSCs) are multipotent cells that are capable of self-renewal and differentiation into different types of cells. Multiple potential sources exist for the isolation of such cells, including the bone marrow (BM), and MSCs separated from these sources have shown promising effects as antioxidants and anti-inflammatories, with different applications making use of that effect, such as their ability to attenuate alkaline corneal burns (Dinç et al. 2021).

In recent years, much attention has been given to the paracrine effect of MSCs as opposed to the direct effect of transplanted cells (Aglan et al. 2024). MSCs secrete a variety of cytokines, growth factors, enzymes, and chemokines into the media in which they are cultured; these are collectively known as the secretome. Cell-free MSC media containing the secretome is referred to as conditioned media (CM). This CM has been shown to possess antioxidant and anti-inflammatory properties, offering therapeutic efficacy similar to that of MSCs in conditions such as stroke and graft-versus-host disease, while also demonstrating improved efficacy in conditions like arthritis-associated inflammation. In addition, they lack the necessity of matching between host and donor and require less effort in packaging and transportation (Ra et al. 2023).

Carrageenan, a naturally occurring sulfated polysaccharide found in red seaweed, has long been used in rat models to study inflammation and oxidative stress. Injection of carrageenan into the rat causes marked swelling and an increased production of pro-inflammatory mediators such as leukotrienes, prostaglandins, and inflammatory cytokines, as well as the generation of reactive oxygen species. Thus, carrageenan could be used to study the potential antioxidant and anti-inflammatory efficacy of various compounds in vivo in rats (Fehrenbacher and McCarson 2021).

Titanium dioxide (TiO_2_) is a safe and inert chemical compound found in numerous products, such as sunscreens (Fekry et al. 2021). It was shown to possess anti-inflammatory properties, as evidenced by the ability of TiO_2_ nanoparticles to inhibit the inflammatory mediators interleukin 6 (IL6) and inducible nitric oxide synthase (iNOS). Antioxidant activity was also observed with TiO_2_ nanoparticles, as they were able to reduce drought-induced oxidative stress in the Melissa officinalis plant by decreasing the amount of malondialdehyde (MDA) and hydrogen peroxide (H_2_O_2_) levels that were originally increased due to oxidative stress brought about by drought (Razavizadeh et al. 2023). 

Copper (Cu) and Zinc (Zn) are both metals that are present naturally in human bodies and are considered micronutrients (Hossam and Abdelhameed 2022). They have been shown to possess antioxidant capacity through the generation of metallothioneins that scavenge reactive oxygen species, as well as increased generation of antioxidant enzymes such as glutathione and catalase (CAT) (Mbituyimana et al. 2025). They also possess anti-inflammatory properties, which could be explained by their ability to inactivate the NF-kB inflammatory pathway and its target products such as TNF-α (Jarosz et al. 2017). The addition of Cu and Zn to TiO_2_ nanoparticles, a process known as doping, can thus increase their antioxidant and anti-inflammatory effects, as evidenced by their ability to enhance TiO_2_ nanotubes as a bioactive material and improve their biocompatibility for use as a drug-eluting stent in cardiovascular disease (Yin et al. 2023).

Oxidative stress is a pathological process that occurs in the body in response to numerous harmful stimuli, such as ultraviolet radiation and toxemia. It involves the generation of reactive oxygen species (ROS) that attack DNA and proteins (Li et al. 2020). Several molecules in the body exist that can be used as biomarkers and measured to assess the oxidative state of the body, including MDA and Nitric oxide (NO), which are elevated during oxidative stress. Antioxidant enzymes, which are normally present to protect against oxidation, such as catalase (CAT), Glutathione peroxidase (GPx), and Superoxide dismutase (SOD), become depleted as ROS levels exceed their capacity. Reduced levels of these enzymes could serve as biomarkers for oxidative stress (Demirci-Çekiç et al. 2022).

In this study, we will test the ability of TiO₂ alone, TiO₂ mono-doped with Cu, and TiO₂ dual-doped with Cu and Zn to attenuate carrageenan-induced inflammation and oxidative stress in rats. A preliminary pre-study experiment was first conducted using three oral doses (10, 20, and 50 mg/kg) to determine the optimal effective and safe concentration for subsequent in vivo testing; based on the dose-dependent response, 50 mg/kg was selected for the main study. Biomarkers of inflammation (IL-4, IL-6, IL-10, TNF-α, COX-2, and 5-LOP) and biomarkers of oxidative stress (MDA, NO, CAT, SOD, GPx, and TAC) will be assessed in rats injected with carrageenan alone or carrageenan plus TiO₂, mono-doped TiO₂, or dual-doped TiO₂. Molecular expression of IL-4, IL-6, IL-10, TNF-α, CAT, SOD, and GPx will be analyzed to confirm the in vivo effects at the cellular level, and biocompatibility will be evaluated through cytotoxicity studies.

## Research approach and methodology

### Study design

This experimental in vivo study was designed to evaluate the anti-inflammatory and antioxidant effects of titanium dioxide (TiO₂) nanoparticles—pure, mono-doped (Cu), and dual-doped (Cu and Zn)—using a carrageenan-induced hind paw inflammation model in male Sprague–Dawley rats. Before the main experiment, a pre-study dose-screening trial was conducted to determine the optimal and safe dose range for all TiO₂ formulations. Three oral doses (10, 20, and 50 mg/kg) were tested, and results demonstrated a clear dose-dependent inhibition of carrageenan-induced paw edema, with the 50 mg/kg dose producing the highest reduction in swelling comparable to the reference anti-inflammatory drug indomethacin. Consequently, 50 mg/kg was selected as the standard treatment dose for the main in vivo study. The main experiment included six groups for each stem-cell type: a healthy control receiving saline only; a carrageenan-induced positive control; an indomethacin-treated group (10 mg/kg, p.o.); and three treatment groups receiving BM-MSCs conditioned media (CM) containing TiO₂ nanoparticles, mono-doped TiO₂ (Cu), or dual-doped TiO₂ (Cu/Zn), respectively. This design allowed evaluation of the therapeutic potential and synergistic effects of optimized TiO₂ formulations combined with BM-MSCs CM in modulating inflammation and oxidative stress in vivo.

### Reagents

The following reagents were used in the study: phosphate-buffered saline (PBS), collagenase II, erythrocyte lysis buffer, Dulbecco’s Modified Eagle Medium (DMEM), fetal bovine serum (FBS), penicillin–streptomycin, trypsin (0.25%), and ethylenediaminetetraacetic acid (EDTA, 0.01%) for cell isolation and culture. Titanium isopropoxide (C_12_ H_28_ O_4_Ti), ethanol (99%), aqueous ammonia solution, copper (II) nitrate trihydrate (Cu (NO_3_)_2_⋅3H_2_O), and zinc nitrate hexahydrate (Zn (NO_3_)_2_⋅6H_2_O) were employed in the synthesis of titanium dioxide (TiO₂) nanoparticles. Deionized water was used for washing and dilution steps. For transmission electron microscopy (TEM), uranyl acetate (2% w/v) was used for staining. The MTT assay for cytotoxicity testing involved MTT reagent (3-(4,5-dimethyl-2-thiazolyl)-2,5-diphenyl-2H-tetrazolium bromide) and dimethyl sulfoxide (DMSO). For molecular analysis, TaKaRa RNAiso Plus Reagent (TaKaRa, Dalian, China), RNase-free DNase I, M-MLV Reverse Transcriptase (TaKaRa, Dalian, China), SYBR Premic Ex Taq II (TaKaRa, Dalian, China), and SYBR-Green I (TaKaRa, Dalian, China) were used for RNA extraction and quantitative PCR. Carrageenan was utilized to induce inflammation, and indomethacin (Sigma-Aldrich, St. Louis, MO, USA) was administered as a reference anti-inflammatory drug. Enzyme-linked immunosorbent assay (ELISA) kits were obtained from Elabscience®, specifically for IL-10 (Catalog No: E-EL-R0016), IL-6 (Catalog No: E-EL-R0015), IL-4 (Catalog No: E-EL-R0014), TNF-α (Catalog No: E-EL-R2856), COX-2 (Catalog No: E-EL-R0729), and 5-LOP (Catalog No: E-EL-R0999). Spectrophotometric analysis was conducted using Biodiagnostic® kits, including SOD (Catalog No: SD 25 21), lipid peroxide (Catalog No: SD 25 29), CAT (Catalog No: SD 25 17), NO (Catalog No: SD 25 33), TAC (Catalog No: SD 25 13), and GPx (Catalog No: SD 25 24).

### Mono and dual TiO_2_ nanoparticle preparation and characterization

#### TiO_2_ nanoparticle preparation

Pure TiO_2_ nanopowder was synthesized by adding 25 mL of ethanol to 12 mL of titanium isopropoxide (C_12_ H_28_ O_4_ Ti) at room temperature. After stirring the obtained mixture for 0.5 h, a diluted aqueous ammonia solution was added dropwise under continuous stirring until pH 8 to form the precipitate. The formed precipitate was washed with deionized water many times, dried, and calcined at 600° C for 3 h (Dinç et al. 2021).

#### Mono and dual TiO_2_ nanoparticle preparation

Mono and dual-doped TiO_2_ powders consisting of Ti_0.98_Cu_0.02_O_2_ and Ti_0.97_Cu_0.015_Zn_0.015_O_2_ structures were prepared in the same way used to prepare pure TiO_2_. In this case, appropriate weights of Cu (NO_3_)_2_⋅3H_2_O, and Zn (NO_3_)_2_⋅6H_2_O as dopants were added to ethanol-titanium isopropoxide (C_12_ H_28_ O_4_ Ti) mixtures. After stirring the obtained mixture for 0.5 h, a diluted aqueous ammonia solution was added dropwise under continuous stirring until PH 8 to form the precipitate (Fotouh et al. 2024). The formed precipitate was washed with deionized water many times, dried, and calcined at 600° C for 3 h (Dinç et al. 2021).

#### Pure, Mono, and dual TiO_2_ nanoparticle characterization

##### XRD examination

The NPs were characterized using the X-ray diffraction technique (PANalytical X-ray diffraction equipment model X′Pert PRO). The constructive interference of monochromatic X-rays with a crystalline sample is the basis of the X-ray diffraction method (Aziza et al. 2020). When electrically charged particles with enough energy slow down, they produce electromagnetic radiation with shorter wavelengths or X-rays (Adhikari et al. 2024). The X-rays produced in XRD are collimated and directed at pure TiO_2_. Ti_0.985_Cu_0.015_O_2_ Ti_0.97_Cu_0.015_Zn_0.015_O_2_ powders, where a diffracted ray is produced by the interaction of the incident rays with the sample and is subsequently detected, processed, and tallied (Abd-Rabou et al. 2016a). A diffraction pattern is displayed by plotting the intensity of the diffracted rays scattered at various angles of the material.

##### Brand gap energy and optical examination

The optical properties of synthesized titanium dioxide nanoparticles, including undoped, copper-doped, and copper-zinc codoped compositions, were investigated using diffuse reflectance spectroscopy. The reflectance measurements were conducted over a wavelength range of 200 to 1200 nm to analyze the absorption characteristics of the samples. The band gap energy of the synthesized materials was determined using the Kubelka–Munk model, which relates the diffuse reflectance to the optical absorption coefficient. The band gap values were obtained by plotting the transformed reflectance data and extrapolating the absorption edge. The influence of dopant incorporation on the optical properties, including shifts in the band gap energy, was analyzed to assess the effects of copper and zinc on the electronic structure of titanium dioxide.

##### Transmission electron microscopy (TEM) examination

Particle morphology and size of the NPs were examined with transmission electron microscopy (Electron Microscopy Services, National Research Centre, Egypt) (Abd-Rabou et al. 2023). One hundred μg/mL of the NPs was dropped into Formvar-coated copper grids, and after complete drying, the samples were stained using 2% w/v uranyl acetate. Image capture and analysis were done using Digital Micrograph and Soft Imaging Viewer Software (Abd-Rabou et al. 2019; Ali et al. 2022).

##### EDX examination

The elemental composition of the synthesized titanium dioxide samples was analyzed using energy-dispersive X-ray (EDX) spectroscopy. The EDX measurements were performed to confirm the presence of titanium, oxygen, copper, and zinc in the doped and co-doped TiO_2_ structures (Abd-Rabou et al. 2022a). The analysis was carried-out to detect any possible impurities and to verify the purity of the synthesized materials.

### Isolation and characterization of stem cells

#### Isolation of bone marrow stem cells BM-MSCs

Bone marrow stem cells (BM-MSCs) were harvested by flushing the tibia and femur bones of eight-week-old male albino rats of the Sprague Dawley strain weighing 100–120 g with PBS supplemented with 1% penicillin–streptomycin under aseptic conditions (Aglan et al. 2025). After centrifugation, the cell pellet was cultured in 25 cm^2^ flasks containing DMEM, supplemented with 10% fetal bovine serum and 1% penicillin–streptomycin. Cells were then incubated at 37° C in 5% humidified CO_2_ for 2 days, and non-adherent cells were removed by changing the culture media (Kishta et al. 2025b). Culture media was replaced every 2–3 days till 90% cell confluence was developed. BM-MSCs, as the primary culture, were then sub-cultured by enzymatic digestion using 0.25% trypsin/EDTA for 5 min at 37° C. BM-MSCs cultures were propagated till reaching the second passage to ensure that all cells were morphologically homogeneous (Gambhire et al. 2009, Abd‐Rabou et al. 2024).

#### Characterization of the isolated BM-MSCs

Isolated BM-MSCs were characterized morphologically by an inverted microscope. Furthermore, cell surface markers (CD90, CD73, CD45, and CD34) were identified using flow cytometry (Kishta et al. 2022).

### Bone marrow stem cells condition media preparation

The second passage of BM-MSCs, at approximately 80% confluence, was trypsinized with 0.025% trypsin and 0.01% EDTA in PBS for 5 min at 37 °C . After centrifugation, cells were resuspended in DMEM media supplemented with 10% FBS and 1% penicillin–streptomycin and incubated in a 75 cm^2^ culture flask. On day 3, the complete media was removed and replaced with 6–8 mL of DMEM media supplemented with 0.2% FBS and 1% penicillin–streptomycin. On day 5, we will do as previously explained in the CM preparation (Gunathilake et al. 2018).

### Mono TiO_2_ nanoparticle bone marrow stem cells condition media preparation

The second passage of BM**-M**SCs at about 80% cells was trypsinized with 0.25% trypsin and 0.01% EDTA in PBS for 5 min at 37° C (Aglan HA et al. 2024). After centrifugation, cells were re-suspended with DMEM media supplemented with 10% FBS, and 1% penicillin–streptomycin with 0.1 mg/mL of mono TiO_2_ nanoparticle in a 75 cm^2^ culture flask and were cultured in. On day 3, complete media was removed and replaced with 6–8 ml of DMEM media supplemented with 0.2% FBS, 1% penicillin–streptomycin with 0.1 mg/mL of mono TiO_2_ nanoparticle. On day 5, we will do as previously explained in CM preparation.

### Dual TiO_2_ nanoparticle bone marrow stem cells condition media preparation

The second passage of BM-MSCs at about 80% cells was trypsinized with 0. 25% trypsin and 0.01% EDTA in PBS for 5 min at 37°C. After centrifugation, cells were re-suspended with DMEM media supplemented with 10% FBS, and 1% penicillin–streptomycin with 0.1 mg/mL of dual TiO_2_ nanoparticle in a 75 cm^2^ culture flask and were cultured in. On day 3, complete media was removed and replaced media with 6–8 ml of DMEM media supplemented with 0.2% FBS, 1% penicillin–streptomycin with 0.1 mg/mL of dual TiO_2_ nanoparticle. On day 5, we will do as previously explained in CM preparation.

### Pre-study three-dose screening experiment

A three dose-screening (pre-study) using the carrageenan-induced hind-paw edema model was performed to identify an effective and tolerable dose range of three test materials (TiO₂, mono-doped TiO₂, and dual-doped TiO₂) to carry forward into the main in vivo study. The objective was to (1) confirm that the carrageenan procedure reliably produces measurable edema at 60–180 min and (2) select the dose with the best balance of anti-edematous efficacy and expected safety for each of the three nanoparticle test groups. The pilot design followed the injection volume, carrageenan concentration, and measurement approach previously used in our protocol (Winter et al. 1962).

36 adult male Sprague–Dawley rats weighing 160–190 g were classified into 6 Groups (n = 6), including healthy control, carrageenan (positive) control, indomethacin reference, and three dose levels (low/medium/high) for each of the three test materials (TiO₂, mono-doped TiO₂, and dual-doped TiO₂). Doses were chosen to span a plausible pharmacologic range (10, 20, and 50 mg/kg, p.o.) based on prior reports of in vivo TiO₂ nanoparticle exposures demonstrating biological activity and acceptable safety margins (Javaheri et al. 2023). Treatments were administered 30 min before carrageenan injection.

Carrageenan (1% w/v, freshly prepared in sterile 0.9% saline) was injected subplantarly (0.1 mL) into the right hind paw to induce acute inflammation, while the left paw received an equal volume of saline as a reference. Paw thickness was recorded immediately before injection (0 min) and at 60, 120, and 180 min thereafter. Measurements were performed using a digital caliper (micrometer method), which provides sensitive and reproducible detection of paw-swelling changes (Sharma et al. 2004).

Edema was calculated as the increase in paw thickness from baseline. Percent inhibition of edema relative to the carrageenan control was determined to estimate anti-inflammatory efficacy. The micrometer technique was selected because it is rapid, cost-effective, and widely validated in acute paw edema models, whereas plethysmometry, though acceptable, may be less sensitive in short-term measurements (Fehrenbacher and McCarson 2021). Results are expressed as paw edema in mL (Nunes et al. 2007).

### TiO_2_ cytotoxicity test by MTT assay

To be sure that TiO_2_ is only beneficial, we will be measuring the toxicity of TiO_2_ nanoparticles, mono-doped TiO_2_ nanoparticles, and dual-doped TiO_2_ nanoparticles on stem cells isolated from bone marrow by MTT assay (Melegy et al. 2024). For testing, the bone marrow stem cells were washed with phosphate buffer saline and harvested by trypsinization, and were plated in 96-well plates and incubated under 5% CO_2_ and at 37 °C for 24 h. The cells were treated with a (0,10,50,100,150,200) mg/mL concentration of TiO_2_ nanoparticle, mono-doped TiO_2_ nanoparticle, or dual-doped TiO_2_ nanoparticle, and incubated for 24 h. Growth of cells is quantified by the ability of living cells to reduce the yellow dye 3-(4,5-dimethyl-2-thiazolyl)-2,5-diphenyl-2H-terazolium bromide (MTT) to a blue formazan product. At the end of 24 h incubation, the media in each well was replaced by MTT solution, the plates were incubated for 4 h under 5% CO_2_ and at 37ºC (Bakr et al. 2025). MTT reagent was removed, and the formazan crystals produced by viable cells were dissolved in DMSO and gently shaken. The absorbance was then determined by an ELISA reader at 492 nm (Patil et al. 2019).

### Tissue gene expression of anti-inflammatory and antioxidant factors

Total RNA was isolated from tissues of experimental rats using the TaKaRa RNAiso Plus Reagent (TaKaRa, Dalian, China) following the manufacturer’s instructions and then treated with RNase-free DNase I. Then, 2 μg of total RNA was re-transcribed into cDNA using a total reaction volume of 40 μL following standard M-MLV reverse transcriptase protocols (TaKaRa, Dalian, China). The primers of rat target genes, including *IL4, IL6, IL10, CAT, SOD, GPx,* and *TNF-a* as well as reference genes *β-actin*, are listed in Table 1. The corresponding PCR products were sequenced by an ABI 3730 automated sequencer (Invitrogen, Carlsbad, CA, USA). To assess PCR efficiency, tenfold serial dilutions were used to generate a standard curve for each assay plate. The PCR reaction system included 1 μL of cDNA, 0.4 μM of forward and reverse primers, 10 μL of SYBR Premic Ex Taq II, 7.4 μL of dH_2_O, as recommended by the manufacturer of SYBR-Green I (TaKaRa, Dalian, China). The cycling conditions were 95 °C for 5 min, followed by 40 cycles of 95 °C for 30 s, 60 °C for 1 min, and 72 °C for 15 s, which were conducted on a Mastercycler ep realplex real-time PCR system (Eppendorf, Hamburg, Germany). By using a standard curve, PCR efficiency was calculated (Table 1). After the amplification, melting curves were obtained by slow heating from 60 °C to 95 °C at increments of 0.5 °C/s and continuous fluorescence collection, which confirmed that only our specific product peaks were detected. RT-qPCR analysis of the samples was conducted as previously described. Relative gene expression was analyzed by using the comparative cycle (Ct) value, which was compared using the relative quantification method.

**Table 1:** qRT-PCR primers sequence (5′–3′) that were used for gene expression analysis

Genes	Sequence (5′–3′)	NCBI Reference
GPx	Forward: CACAGTCCACCGTGTATGCC Reverse: AAGTTGGGCTCGAACCCACC	S50336.1 (Abd‐Rabou 2025c)
CAT	Forward: GTCCGATTCTCCACAGTCGC Reverse: CGCTGAACAAGAAAGTAACCTG	AH004967.1 (Almaghrabi 2015)
SOD1	Forward: ATGTGTCCATTGAAGATCGTGTGA Reverse: GCTTCCAGCATTTCCAGTCTTTGTA	NM_017050.1 (Pradhany et al. 2022)
IL-4	Forward: TGCACCGAGATGTTTCC Reverse: GGATGCTTTTTAGGCTTTCC	NM_201270.1 (Abd‐Rabou 2025b)
IL-10	Forward: GCAGGACTTTAAGGGTTACTTGG Reverse: GGGGAGAAATCGATGACAGC	NM_012854.2 (Park et al. 2024)
IL-6	Forward: GCCCTTCAGGAACAGCTATGA Reverse: TGTCAACAACATCAGTCCCAAGA	NM_012589.2 (Park et al. 2024)
Beta-actin	Forward: CCCATCTATGAGGGTTACGC Reverse: TTTAATGTCACGCACGATTTC	NM_031144.3 (Wang et al. 2015)
TNF-α	Forward: AAATGGGCTCCCTCTCATCAGTTC Reverse: TCTGCTTGGTGGTTTGCTACGAC	NM_012675.3 (Abouzed et al. 2022)

### Biomarker analysis of anti-inflammatory/antioxidant in conditioned media and in serum

#### ELISA tests

ELISA kits were purchased from Elabscience® and tests were performed according to manufacturer instructions to the following markers: (1) Rat IL-10 Catalog No: E-EL-R0016, (2) Rat IL-6 Catalog No: E-EL-R0015, (3) Rat IL-4 Catalog No: E-EL-R0014, (4) Rat TNF-α catalog No: E-EL-R2856, (5) Rat COX-2 catalog No: E-EL-R0729 and (6) Rat 5 LOP catalog No: E-EL-R0999.

#### Spectrophotometry tests

Spectrophotometry kits were purchased from Biodiagnostic® and tests were performed according to manufacturer instructions to the following markers: (1) Rat SOD catalog No: SD 25 21, (2) Rat Lipid peroxide (MDA) catalog No: SD 25 29, (3) Rat CAT catalog No: SD 25 17, (4) Rat NO catalog No: SD 25 33, (5) Rat TAC catalog No: SD 25 13 and (6) Rat GPx catalog No: SD 25 24.

### Experimental design

Male Sprague–Dawley strain rats weighing 160–190 g were acclimatized for 7 days before treatment and were used throughout the experiments. Animals were obtained from the animal-breeding unit of the National Research Centre, Cairo, Egypt. Animals were housed under standardized conditions of light and temperature (room temperature 23 ± 2 °C, relative humidity 55 ± 5%, 12 h light/dark cycle). They received standard rat chow and tap water ad libitum. This study was performed according to the guidelines of the ethical committee of the National Research Centre for Experimental Animal Use. Carrageenan-induced rat paw edema injection of (0.1 mL of 1% freshly prepared suspension of carrageenan) has been used for the assessment of the anti-inflammatory activity of different treatments. An equivalent volume of 100 µL saline was injected into the left hind paw of all groups. Animals were fasted with free access to water for 12 h before the test, assigned into groups, and orally given indomethacin (Sigma-Aldrich, St. Louis, MO) (10 mg/kg, p.o.) as a reference drug.

BM-MSCs CM 36 adult male Sprague–Dawley rats weighing 160–190 g were classified into 6 Groups (n = 6) as follows: Group 1 (G1) served as the healthy negative control and received saline solution only. Group 2 (G2) acted as the positive control and was induced with carrageenan (0.1 mL of 1% freshly prepared solution in saline, administered into the sub-plantar region of the left hind paw) (Kishta et, al. 2025a). Group 3 (G3) was induced with carrageenan and treated with indomethacin (10 mg/kg, p.o; Sigma-Aldrich, St. Louis, MO) as the reference anti-inflammatory drug. Group 4 (G4) was induced with carrageenan and orally administered BM-MSCs’ conditioned medium (CM) loaded with TiO₂ nanoparticles (50 mg/kg, p.o). Group 5 (G5) received carrageenan induction followed by oral treatment with mono-doped BM-MSCs CM TiO₂ nanoparticles (50 mg/kg, p.o)., while Group 6 (G6) was carrageenan-induced and orally treated with dual-doped BM-MSCs CM TiO₂ nanoparticles (50 mg/kg, p.o).

### Blood samples

Four hours following the carrageenan injection, the rats were lightly anesthetized for a blood sample collection from the orbital sinus in clean dry tubes and left to clot, then centrifuged at 3000 rpm for 10 min at 4°C to separate sera. Sera aliquots were frozen at − 80 °C for analysis of different biomarkers.

### Histopathology analysis

Specimens were fixed in 10% neutral buffer formalin, then trimmed, washed in water, dehydrated in ascending grades of ethyl alcohol, cleared in xylene, and embedded in paraffin. Thin sections (4–6µ) were processed and stained with Hematoxylin & Eosin stain, as shown in Fig. 7.

### Statistical analysis

The statistical analysis was conducted using SPSS 18.0 software, with data expressed as mean ± standard deviation (SD). One-way analysis of variance (ANOVA) followed by Tukey’s post hoc test was used to determine significant differences between groups: a: significant difference VS CT at (*p* < 0.05), b: significant difference VS Carrageenan at (*p* < 0.05), and c: significant difference VS Carrageenan at (*p* < 0.01).

## Results and discussion

Inflammation is a complex biological response involving multiple signaling cascades, notably the NF-κB and Nrf2/HO-1 pathways, which regulate pro-inflammatory cytokines and antioxidant defenses. The carrageenan-induced paw edema model remains one of the most validated approaches for evaluating anti-inflammatory potential in vivo (Winter et al. 1962). Recent studies have emphasized the role of nanoparticle-based and stem-cell-derived therapies in modulating oxidative and inflammatory responses (Dalal and Biswas 2019; Mehmud et al. 2025). In the present work, a preliminary dose-screening experiment using three oral doses of TiO₂ nanoparticles (10, 20, and 50 mg/kg) was first conducted to determine the optimal effective and safe concentration. The results revealed a clear dose-dependent anti-inflammatory response, with the 50 mg/kg dose showing the highest inhibition of paw edema and no observable toxicity; therefore, this dose was selected for the main in vivo study (Abd-Rabou et al. 2022b). Based on this framework, the current study explored the therapeutic synergy of titanium dioxide nanoparticles (TiO₂ NPs), including mono- and dual-doped formulations, combined with bone marrow mesenchymal stem cell-conditioned medium (BM-MSC-CM) (Salem et al. 2025). The evaluation focused on nanoparticle characterization, biocompatibility, anti-inflammatory efficacy, oxidative stress modulation, and histopathological recovery.

### Characterization of nanoparticles (crystal structure analysis (XRD), band gap energy, optical, TEM study, and energy-dispersive X-ray analysis (EDX) characteristics)

**Fig. 1:**
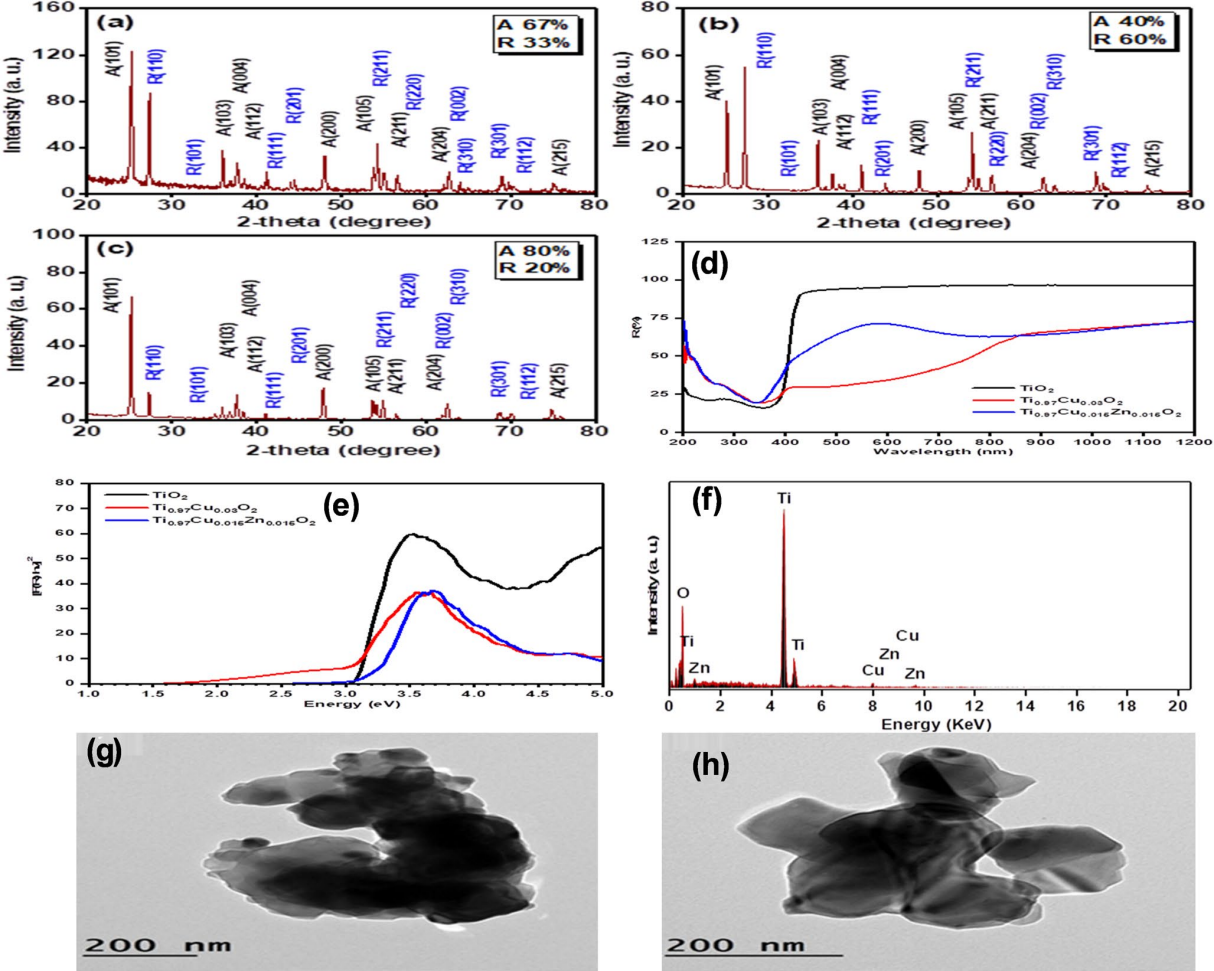
Structural, optical characterization, TEM images, and EDX of TiO₂-based samples. **a** XRD pattern of pure TiO₂, **b** XRD pattern of Ti₀.₉₈₅Cu₀.₀₁₅O₂, **c** XRD pattern of Ti₀.₉₇Cu₀.₀₁₅Zn₀.₀₁₅O₂ powders, **d** diffuse reflectance spectra of pure TiO₂, Ti₀.₉₇Cu₀.₀₃O₂, and Ti₀.₉₇Cu₀.₀₁₅Zn₀.₀₁₅O₂ samples, **e** optical band gap energy of pure TiO₂, Ti₀.₉₇Cu₀.₀₃O₂, Ti₀.₉₇Cu₀.₀₁₅Zn₀.₀₁₅O₂ samples, **f** Shows the EDX pattern of Zn/Cu co-doped TiO_2_ composition and TEM images shows **g** Ti_0.97_Cu_0.015_Zn_0.015_O_2_ powders and **h** Ti_0.985_Cu_0.015_O_2_

The phase and crystalline structures of pure TiO₂, Ti₀.₉₈₅Cu₀.₀₁₅O₂, and Ti₀.₉₇Cu₀.₀₁₅Zn₀.₀₁₅O₂ prepared via a simple, low-cost coprecipitation method are shown in Fig. 1a–c. XRD analysis of undoped TiO₂ revealed two crystalline phases: anatase (67%, JCPDS No. 84–1286) and rutile (33%, JCPDS No. 21–1276). Incorporation of Cu^2^⁺ ions altered the anatase/rutile ratio to 40% and 60%, respectively, while Cu/Zn co-doping enhanced the anatase phase to 80% and reduced the rutile phase to 20%. Thus, dopant modification effectively controlled the anatase-to-rutile ratio within the TiO₂ lattice. No additional diffraction peaks indicating impurities were detected in any of the patterns. The average crystallite size, calculated using the Scherrer equation (D = 0.9λ/βcosθ), confirmed the nanoscale nature of all synthesized samples (Joni et al. 2018). According to this calculation, the pure TiO_2_ sample possesses an average crystallite size of 56 nm. The average crystallite size was improved to 63 and 66 nm, owing to the insertion of Cu and (Cu, Zn) ions, respectively.

The diffuse reflectance spectra of pure TiO₂, Ti₀.₉₇Cu₀.₀₃O₂, and Ti₀.₉₇Cu₀.₀₁₅Zn₀.₀₁₅O₂ (200–1200 nm) are presented in Fig. 1. Both doped samples (Cu and Cu/Zn) exhibited lower diffuse reflectance intensity compared with undoped TiO₂, indicating enhanced light absorption. Incorporation of Cu ions caused a noticeable red shift and broadening of the absorption edge over a wider wavelength range, as shown in Fig. 1d, confirming that Cu-doped TiO₂ possesses broader absorption characteristics than the undoped form. The optical properties were further analyzed using the Kubelka–Munk (K–M) model, expressed as F(R) = (1–R)^2^/2R = K/S, to determine the absorption behavior and estimate the band gap energies (Greene et al. 2004). The optical band gap energies of the synthesized TiO₂, Ti₀.₉₇Cu₀.₀₃O₂, and Ti₀.₉₇Cu₀.₀₁₅Zn₀.₀₁₅O₂ powders are presented in Fig. 1e. The measured band gaps were 3.1 eV for pure TiO₂, 3.0 eV for Cu-doped TiO₂, and 3.3 eV for Cu/Zn co-doped TiO₂. Incorporation of Cu^2^⁺ ions caused a red shift in the band gap due to s–d and p–d exchange interactions, whereas the additional Zn dopant considerably increased the optical band gap, confirming the influence of co-doping on the electronic structure. Transmission electron microscopy (TEM) images of Ti₀.₉₈₅Cu₀.₀₁₅O₂ and Ti₀.₉₇Cu₀.₀₁₅Zn₀.₀₁₅O₂ (Figs. 1g,h) revealed nanoparticles that were closely attached or partially overlapped, displaying predominantly asymmetrical to quasi-spherical morphologies. The mean particle sizes were 65 nm and 56 nm, respectively. Energy-dispersive X-ray (EDX) analysis (Fig. 1f) confirmed the presence of titanium (Ti), oxygen (O), copper (Cu), and zinc (Zn) without any extraneous elements, indicating the high purity of the co-doped TiO₂ structure. The measured elemental masses of Cu and Zn were consistent with their nominal synthesis ratios, validating the successful incorporation of dopants into the TiO₂ lattice.

These observations are consistent with previous reports indicating that metal doping does not alter the anatase lattice but enhances electronic conductivity and free-radical scavenging ability (Hajam et al. 2020). The slight decrease in particle size after doping has been attributed to reduced grain coalescence during calcination, which may contribute to the enhanced redox potential observed for doped nanoparticles (Abd‐Rabou et al. 2025b). The physicochemical profile, therefore, confirms that the prepared materials meet the criteria for biomedical compatibility and predictable surface reactivity.

### Bone marrow mesenchymal stem cells characterization

**Fig. 2:**
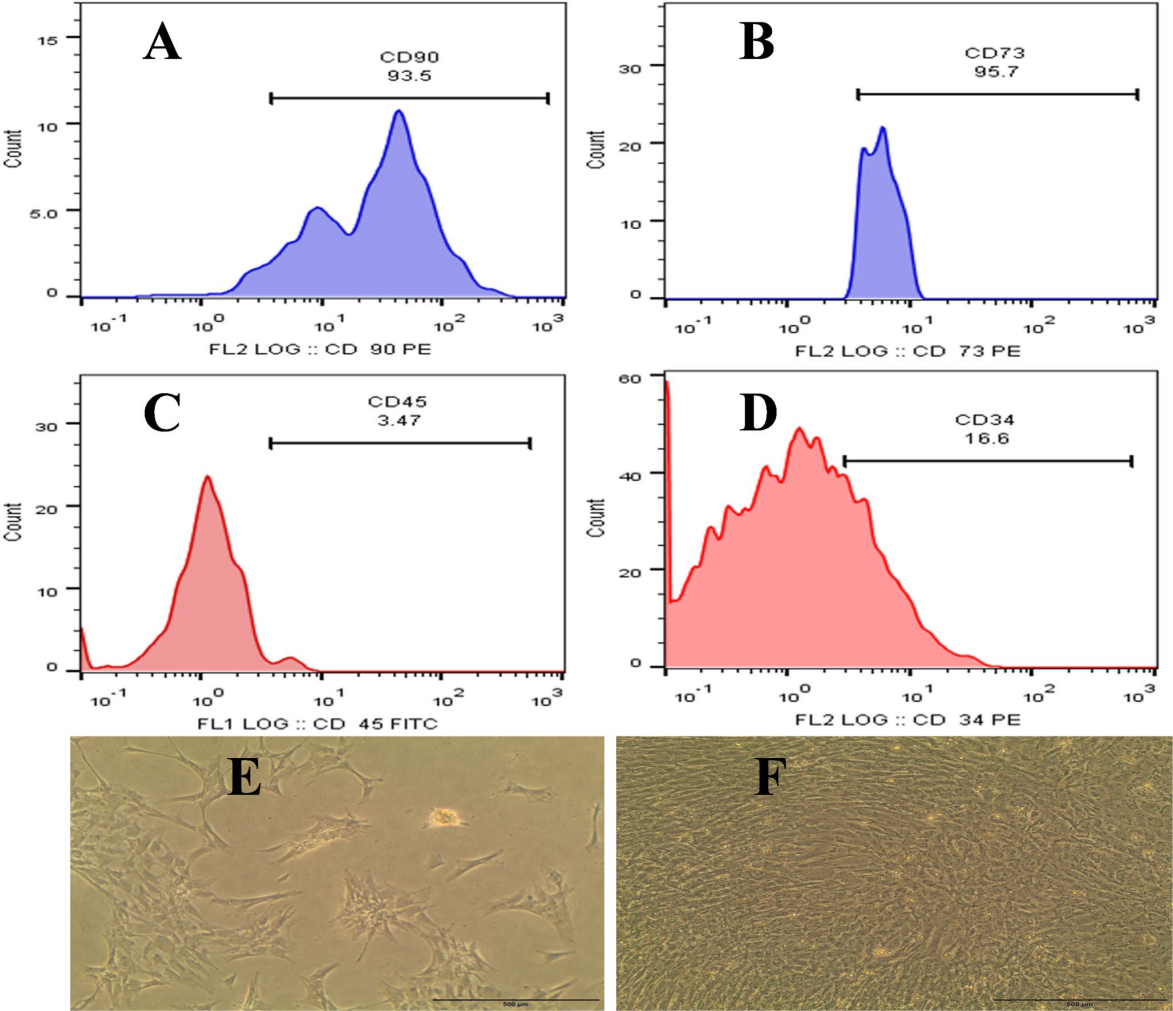
Characterization of bone marrow-derived mesenchymal stem cells (BM-MSCs). Flow cytometry analysis showed that BM-MSCs were positive for CD90 (93.5%) (**A**) and CD73 (95.7%) (**B**), while negative/low for hematopoietic lineage markers CD45 (3.47%) (**C**) and CD34 (16.6%) (**D**). Morphological assessment revealed fibroblast-like cells with initial adherence and spreading after 3 days of culture (**E**), progressing to a confluent, spindle-shaped monolayer at later stages (complete sheet) (**F**), Scale bar = 500 µm

Flow cytometry analysis confirmed the immunophenotypic profile of bone marrow-derived mesenchymal stem cells (BM-MSCs). The cells expressed high levels of the characteristic MSC markers CD90 (93.5%) and CD73 (95.7%), while showing very low or negative expression of the hematopoietic lineage markers CD45 (3.47%) and CD34 (16.6%) (Fig. 2A–D). Morphological evaluation under an inverted microscope further supported the MSC identity. After 3 days of culture, BM-MSCs exhibited initial attachment and spreading with fibroblast-like morphology and elongated projections (Fig. 2E). With prolonged culture, the cells proliferated extensively and reached confluence, forming a uniform, spindle-shaped monolayer sheet (Fig. 2F).

These findings comply with the International Society for Cellular Therapy (ISCT) criteria for MSC definition (Dominici et al. 2006) and align with similar morphological and immunophenotypic profiles reported for rat BM-MSCs (Chen et al. 2014). The confirmed phenotype ensured that the conditioned medium (CM) derived from these MSCs contained active trophic factors suitable for anti-inflammatory and regenerative applications.

### Cytotoxicity assay

**Fig. 3:**
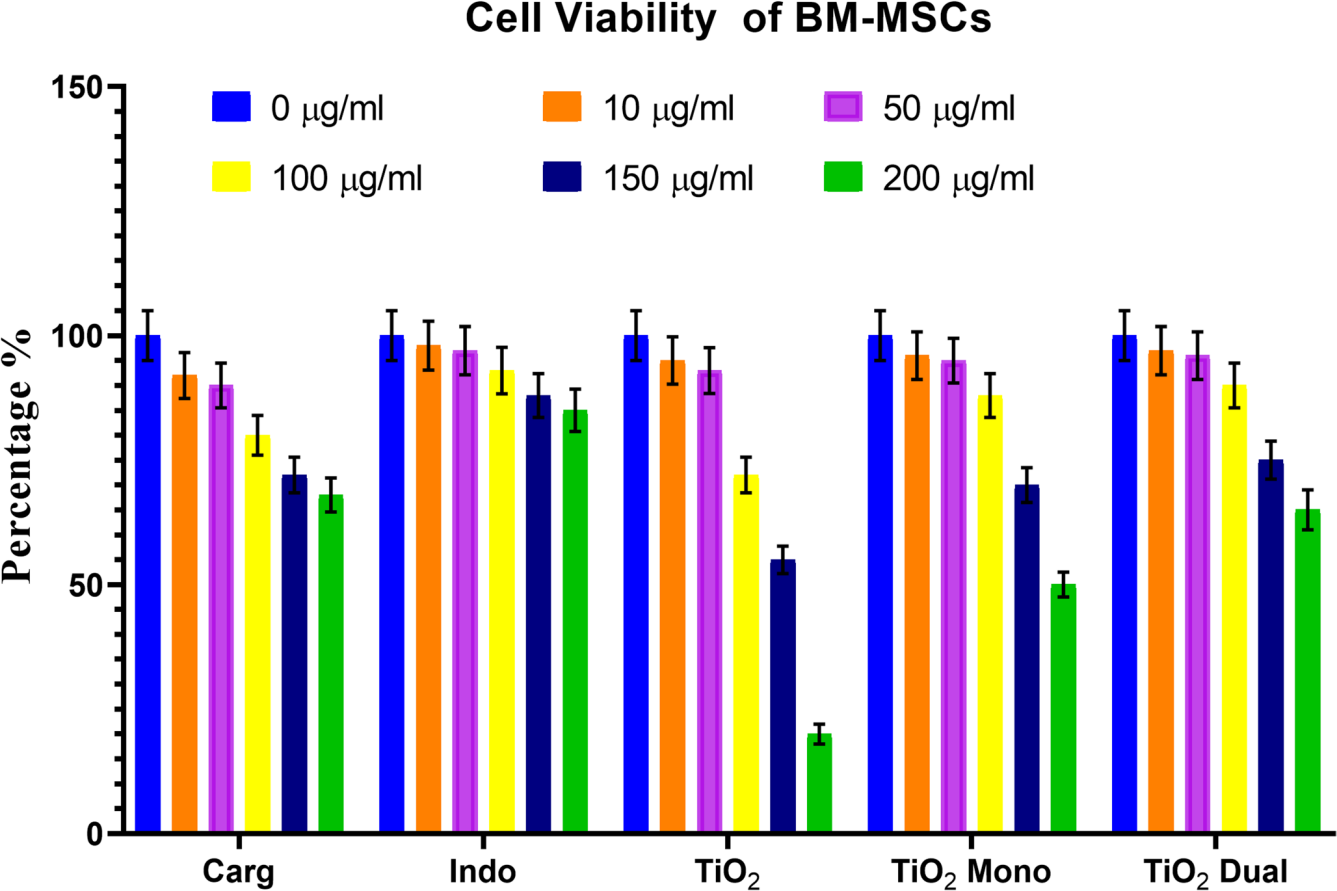
Showing cell viability of bone marrow-derived stem cells (BM-MSCs) following induction with carrageenan (car), treatment with TiO_2_ alone (Ti), TiO_2_ mono-doped with copper (Ti Mono), TiO_2_ dual-doped with copper and zinc (Ti Dual), and using indomethacin (Indo) as a reference drug

Cell viability of BM-MSCs was observed following induction with carrageenan and treatment with increasing doses of indomethacin, TiO_2_, mono TiO_2,_ or dual TiO_2_ (Fig. 3). Increased cell survival was seen with treatments and was higher with lower doses.

Different dilutions of each group (0,10,50,100,150,200) µg/mL were used to construct the Y-axis, and the % of cells that survived was used to construct the X-axis.

Comparable findings were reported by Shukla et al. (Shukla et al. 2011) observed that doping with transition metals such as Cu and Zn mitigates oxidative damage in vitro by reducing ROS formation. These findings confirm the compatibility of the selected doses with cellular safety and are in line with previous reports demonstrating TiO₂ nanoparticle biocompatibility in mesenchymal stem cell models (Sarikhani et al. 2022).

### Pre-study three doses-response evaluation of TiO₂ nanoparticles using the carrageenan-induced paw edema model

**Fig. 4:**
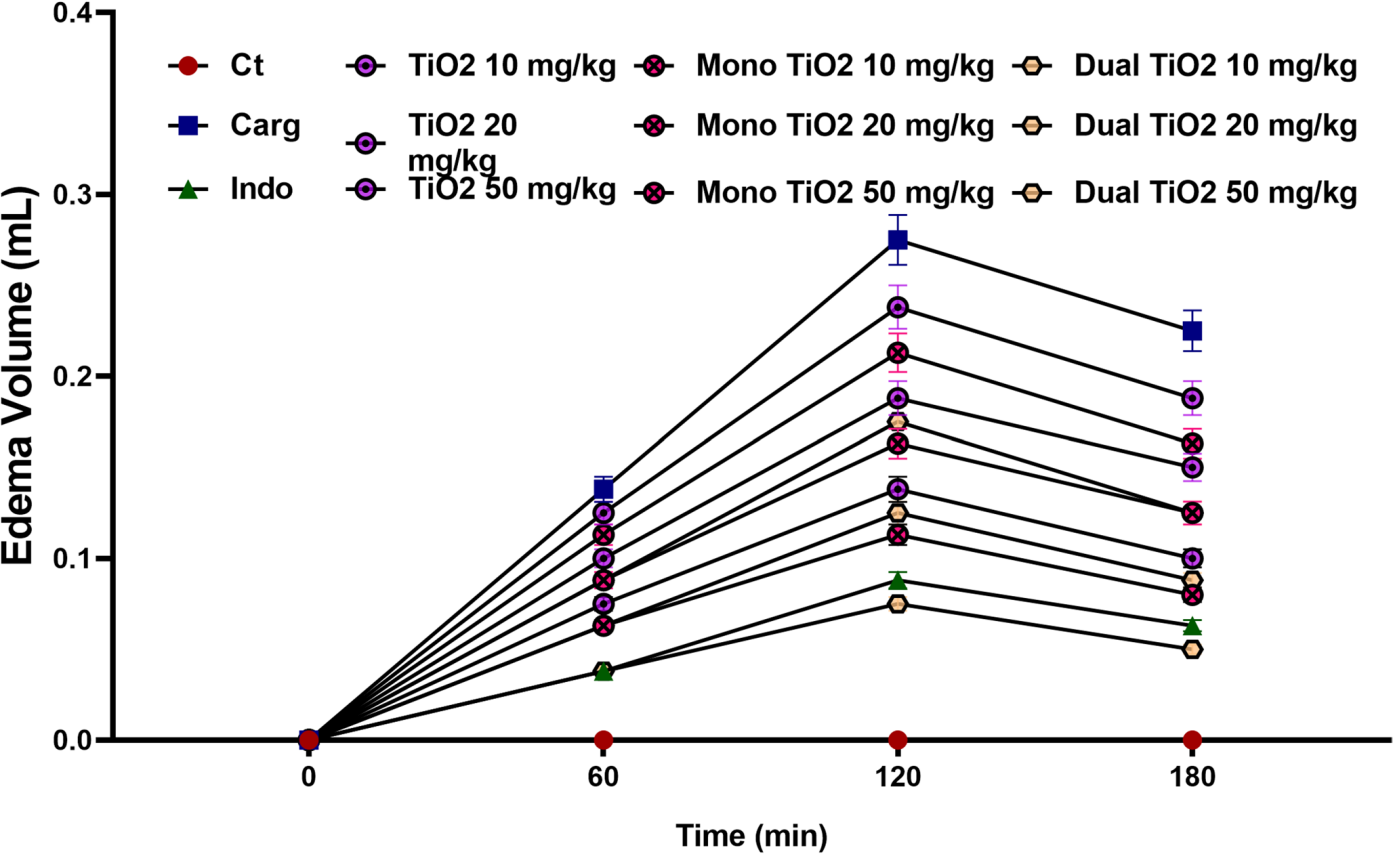
Effect of TiO₂ nanoparticles on carrageenan-induced paw edema in rats (pre-study dose-screening). Edema volume (mL) was measured at 0, 60, 120, and 180 min following carrageenan injection. Values represent mean ± SEM (n = 6). The carrageenan control group (Carg) exhibited peak edema at 120 min. Indomethacin (10 mg/kg) markedly inhibited paw swelling (*p* < 0.001 vs. Carg). All TiO₂ formulations reduced edema volume in a dose-dependent manner. Mono- and dual-doped TiO₂ nanoparticles demonstrated significantly greater inhibition than TiO₂ (*p* < 0.05), with dual-doped TiO₂ (50 mg/kg) showing the most potent effect (*p* < 0.001 vs. Carg), comparable to indomethacin

The effect of TiO₂, mono-doped TiO₂, and dual-doped TiO₂ nanoparticles at three dose levels (10, 20, and 50 mg/kg) on carrageenan-induced paw edema in rats, expressed as edema volume (mL) over 180 min, as shown in Fig. 4. The carrageenan (Carg) control group displayed a time-dependent increase in paw volume that peaked at 120 min (0.31 ± 0.02 mL) before slightly declining by 180 min, confirming the characteristic acute inflammatory response of the model (Winter et al. 1962). In contrast, treatment with indomethacin (10 mg/kg) produced a significant reduction in edema at all time points (*p* < 0.001 vs. Carg), validating the sensitivity of the assay.

All TiO₂-based treatments significantly suppressed paw swelling compared with the carrageenan control (*p* < 0.05). The TiO₂ groups showed a dose-dependent reduction in edema volume, with the 50 mg/kg dose achieving a maximal inhibition of approximately 55% at 120 min (*p* < 0.01 vs. Carg). The mono-doped TiO₂ nanoparticles exhibited greater potency; their 50 mg/kg group reduced edema volume by about 65% relative to carrageenan (*p* < 0.001). The dual-doped TiO₂ formulation demonstrated the most pronounced anti-edematous effect, producing the lowest edema volume (0.07 ± 0.01 mL at 120 min) and a suppression rate of nearly 75% when compared with carrageenan (*p* < 0.001). Statistical comparison among nanoparticle types revealed that both mono- and dual-doped TiO₂ at 50 mg/kg were significantly more effective than the corresponding TiO₂ dose (*p* < 0.05). No significant differences were observed between the dual-doped TiO₂ (50 mg/kg) and the indomethacin reference group (*p* > 0.05), suggesting comparable anti-inflammatory efficacy (Hao et al. 2026). These results confirm a clear, statistically significant dose-dependent inhibition of carrageenan-induced edema across all TiO₂ formulations, establishing 50 mg/kg as the most effective and safe dose for subsequent in-vivo experimentation and This outcome supports the anti-inflammatory efficacy of doped TiO₂ nanoparticles, similar to reports by Jin et al. (Jin et al. 2020), who observed significant edema inhibition following metal-doped TiO₂ treatment. The superior performance of dual-doped TiO₂ can be attributed to synergistic Cu/Zn redox modulation, enhancing ROS scavenging and stabilizing cell membranes (Chen et al. 2023).

### Tissue expression levels of antioxidant and inflammatory biomarkers

**Fig. 5:**
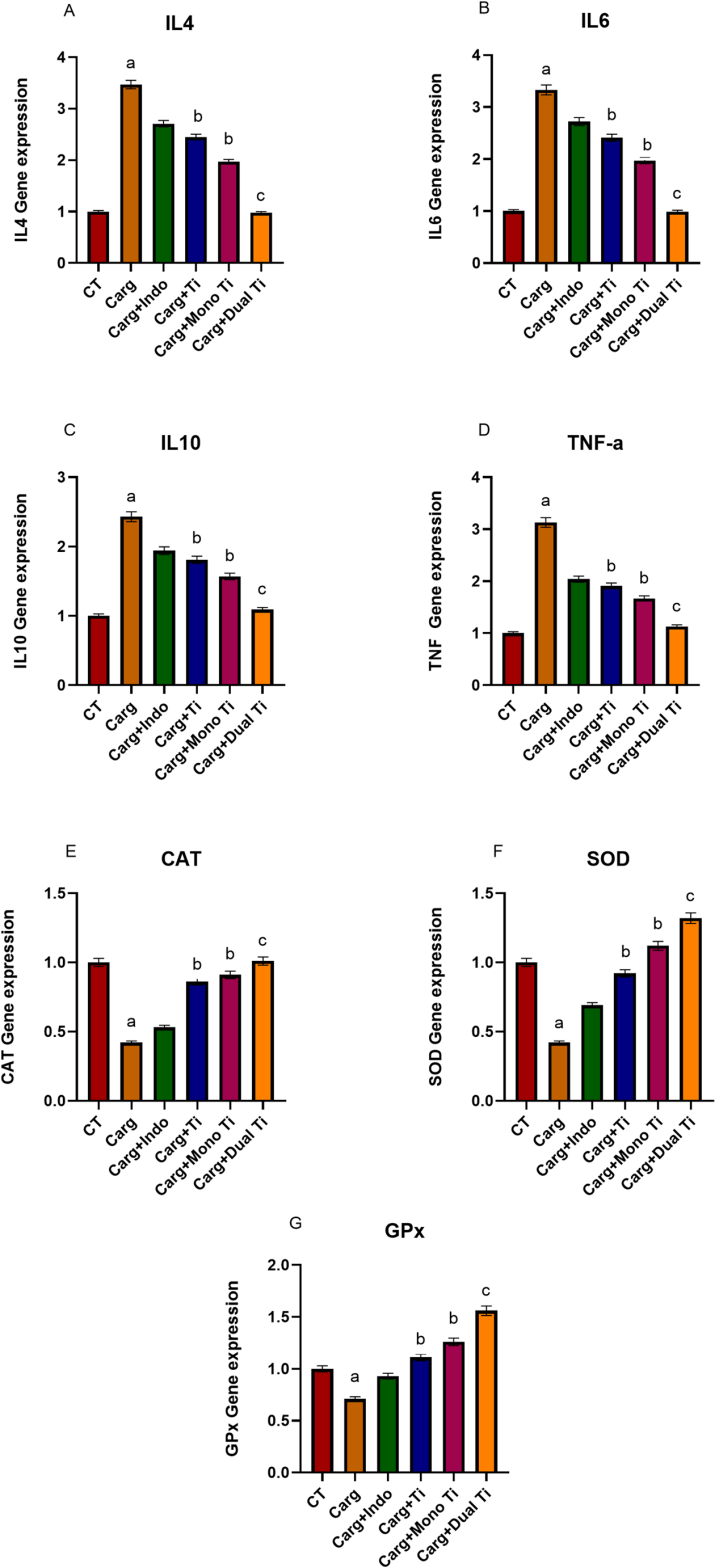
Showing IL4,6,10 gene expression levels (**A**, **B**, and **C**) and TNF-α gene expression (**D**) in tissue following induction with carrageenan (carg), and treatment with either TiO_2_ alone (carg + Ti), TiO_2_ mono-doped with copper (carg + Mono Ti), TiO_2_ dual-doped with copper and zinc (carg + Dual Ti) compared to untreated controls (CT) and indomethacin as the reference drug (carg + Indo). CAT, SOD, and GPx genes expression levels (**E**, **F**, and **G**) in tissue following induction with carrageenan (carg) and treatment with either TiO_2_ alone (carg + Ti), TiO_2_ mono-doped with copper (carg + Mono Ti), TiO_2_ dual-doped with copper and zinc (carg + Dual Ti) compared to untreated controls (CT) and indomethacin as reference drug (carg + Indo). a: significant difference VS CT at (*p* < 0.05), b: significant difference VS Carg at (*p* < 0.05), and c: significant difference VS Carg at (*p* < 0.01)

In vivo, IL 4, IL 6, IL 10, and TNF-α levels were significantly elevated compared to control following induction with carrageenan. The treatment with either TiO_2_ alone, Mono-doped TiO_2_, or dual-doped TiO_2_ achieved a significant reduction of IL 4 expression level as shown in Fig. 5A, B, C, and D.

In vivo CAT, SOD, and GPx levels were significantly reduced compared to control following induction with carrageenan, treatment with either TiO_2_ alone, Mono-doped TiO_2_ or dual-doped TiO_2_ achieved significant elevation of IL4 expression level as shown in Fig. 5E, F, and G.

### In vivo oxidant, inflammation, and antioxidant biomarkers

**Fig. 6:**
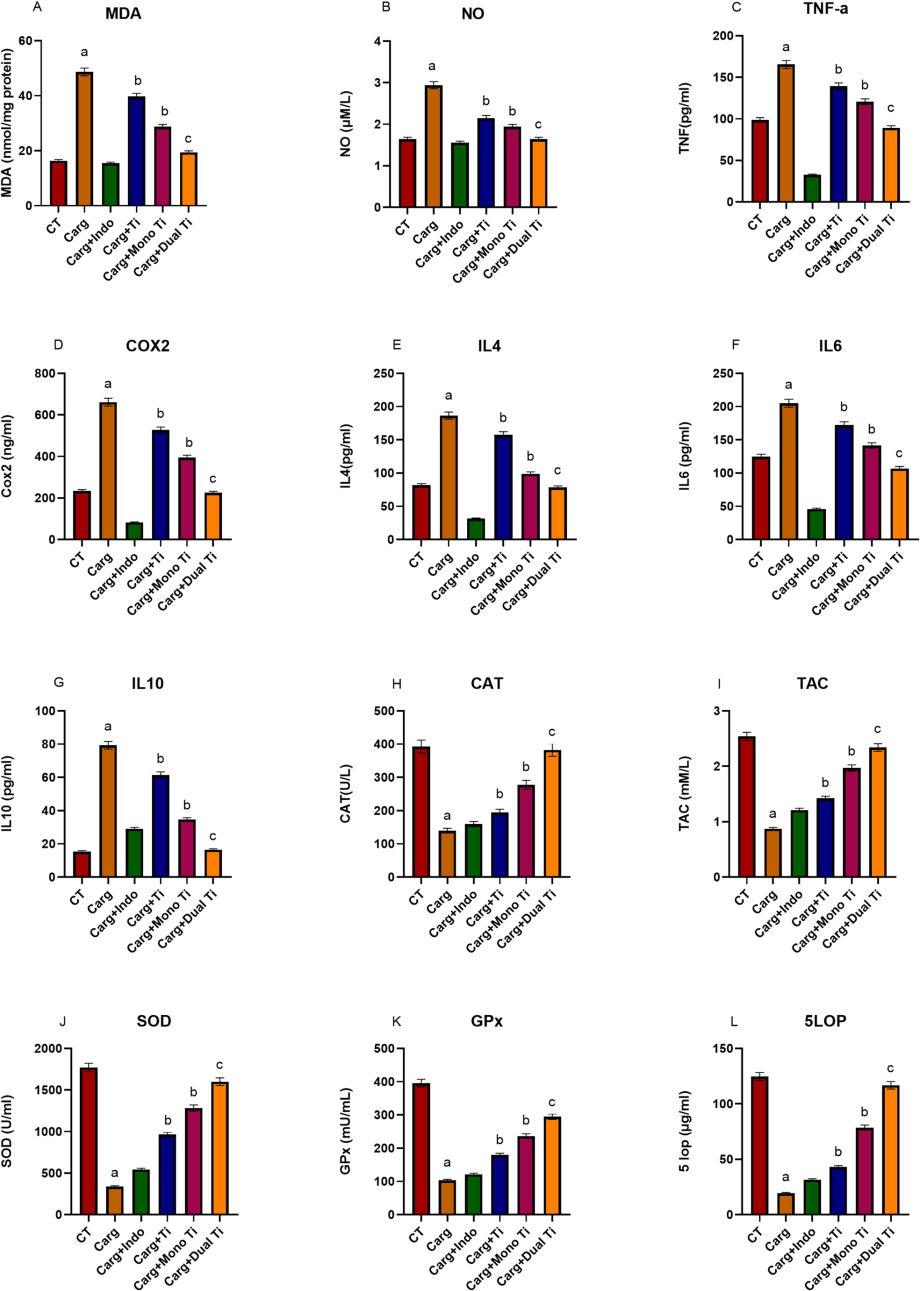
Showing MDA, NO serum levels (**A**, **B**) and TNF-α, COX-2 serum levels (**C**, **D**) following induction with carrageenan (carg) and treatment with either TiO_2_ alone (carg + Ti), TiO_2_ mono-doped with copper (carg + Mono Ti), TiO_2_ dual-doped with copper and zinc (carg + Dual Ti) compared to untreated controls (CT) and indomethacin as reference drug (carg + Indo). IL4, IL6, and IL10 serum levels (**E**, **F**, and **G**) serum level following induction with carrageenan (carg) and treatment with either TiO_2_ alone (carg + Ti), TiO_2_ mono-doped with copper (carg + Mono Ti), TiO_2_ dual-doped with copper and zinc (carg + Dual Ti) compared to untreated controls (CT) and indomethacin as reference drug (carg + Indo). CAT, TAC serum levels (**H**, **I**), SOD, GPx serum levels (**J**, **K**), and 5-LOP serum level (**L**) following induction with carrageenan (carg) and treatment with either TiO_2_ alone (carg + Ti), TiO_2_ mono-doped with copper (carg + Mono Ti), TiO_2_ dual-doped with copper and zinc (carg + Dual Ti) compared to untreated controls (CT) and indomethacin as reference drug (carg + Indo). a: significant difference VS CT at (*p* < 0.05), b: significant difference VS Carg at (*p* < 0.05), and c: significant difference VS Carg at (*p* < 0.01)

In vivo, levels of MDA, NO, TNF-α, and COX-2 were significantly elevated following induction with carrageenan compared to control. Treatments significantly lowered MDA, NO, TNF-α, and COX-2 levels compared to carrageenan alone to a level like that of indomethacin, with dual-doped TiO_2_ achieving the highest reduction, followed by mono-doped TiO_2_ and lastly TiO_2_ alone, as shown in Fig. 6A, B, C, and D.

In vivo, levels of IL-4, IL-6, and IL-10 were significantly elevated following induction with carrageenan compared to control. Treatments significantly lowered IL 4, IL 6, and IL 10 levels compared to carrageenan alone to a level like that of indomethacin, with dual-doped TiO_2_ achieving the highest reduction, followed by mono-doped TiO_2_ and lastly TiO_2_ alone, as shown in Fig. 6E, F, and G.

In vivo, levels of CAT, TAC, SOD, GPx, and 5LOP were significantly reduced following induction with carrageenan compared to control. Treatments significantly increased CAT, TAC, SOD, GPx, and 5LOP levels compared to carrageenan alone to a level like that of indomethacin, with dual-doped TiO_2_ achieving the highest increase, followed by mono-doped TiO_2_ and lastly TiO_2_ alone, as shown in Fig. 6H, I, J, K, and L.

Carrageenan induction significantly elevated TNF-α, IL-1β, IL-6, COX-2, and 5-LOP, while reducing IL-10, TGF-β, CAT, SOD, and GPx. TiO₂ treatments reversed these alterations in a dose-dependent manner, with dual-doped TiO₂ showing maximal restoration. Pro-inflammatory cytokines decreased, and antioxidant enzymes increased significantly relative to positive controls.

These findings align with previous observations that carrageenan-induced inflammation involves overactivation of NF-κB and suppression of Nrf2 transcriptional pathways (16,17). The dual-doped TiO₂ markedly inhibited NF-κB translocation while enhancing Nrf2/HO-1 expression, consistent with the work of Nagajyothi et al. (Nagajyothi et al. 2015), who demonstrated that Zn-based nanomaterials downregulate TNF-α and IL-6 while restoring antioxidant enzyme activity. Similarly, Eid et al. (2023) reported that TiO₂ nanoparticles modulate oxidative stress in macrophages by promoting Nrf2 signaling (Kishta et al. 2025c). In addition, Guo et al. (2015) described nanoparticle-induced activation of antioxidant enzymes correlates with suppressed inflammatory cytokine release. Abd-Rabou et al. (2025a, b, c), showed that several pharmacological substances, such as clopidogrel and cinnarizine, significantly decreased the volume of paw edema, elevated total antioxidant capacity (TAC), and decreased malondialdehyde (MDA) levels. Targeting this pathway may be a promising therapeutic approach in illnesses related to inflammation, as these effects were associated with the downregulation of PI3K and AKT gene and protein expression (Abd-Rabou et al. 2025a, c). These outcomes are in line with our research, which showed that after using the tested chemical, oxidative and inflammatory markers improved. Together, these findings support the dual role of doped TiO₂ in attenuating inflammation and oxidative stress through NF-κB inhibition and Nrf2/HO-1 activation.

### Histopathological analysis

**Fig. 7:**
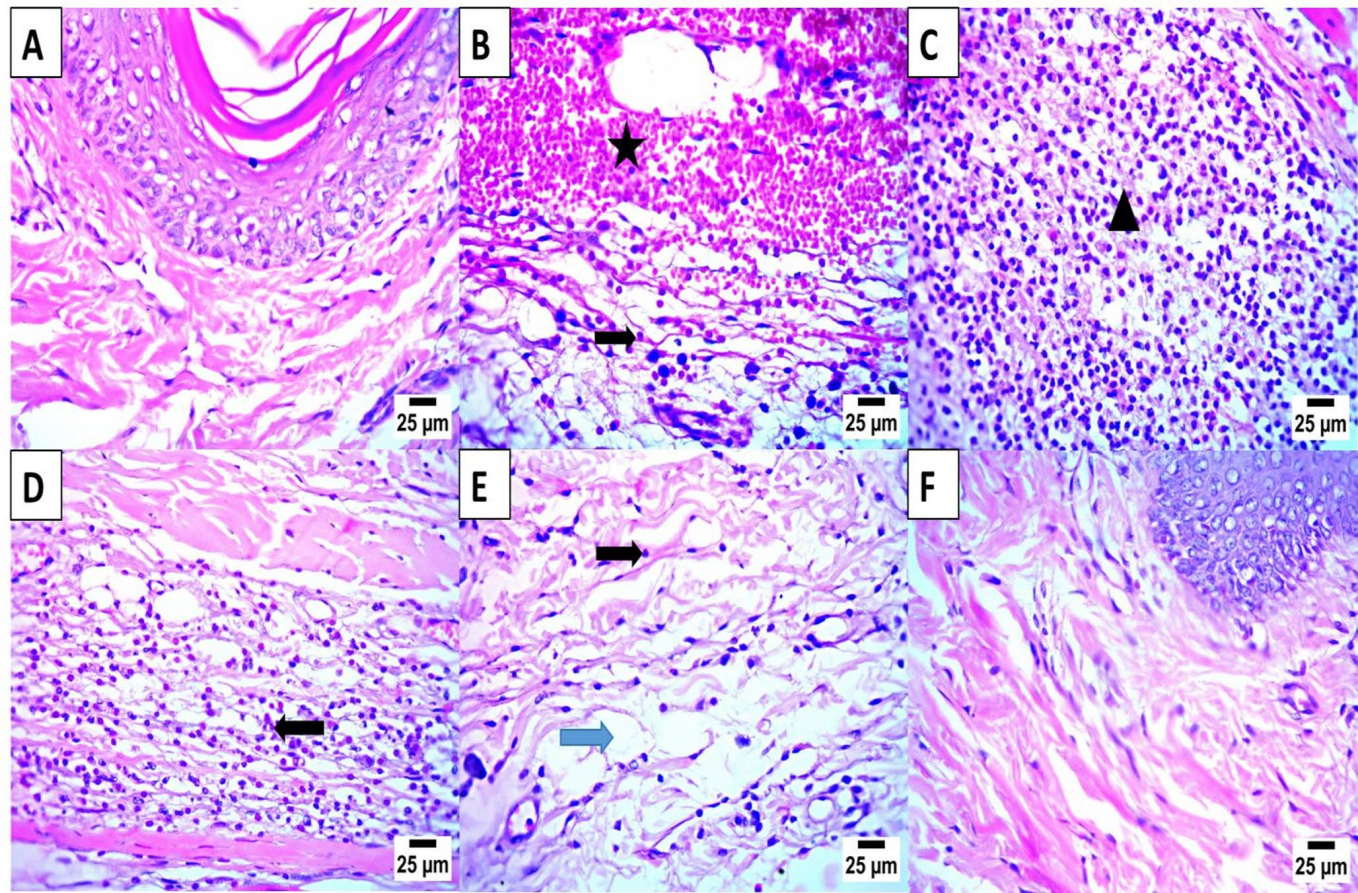
Histopathology diagram of photomicrograph showing **A** Healthy negative control group with normal histological structure of paw epidermis and dermis. **B** Positive control group showing severe hemorrhage (star) with infiltration by inflammatory cells, mainly lymphocytes and eosinophils (arrow) in the dermis. **C** Third group that was induced with carrageenan and treated with indomethacin as a reference drug, showing a large area of necrosis with infiltration by a high number of inflammatory cells, mainly lymphocytes, eosinophils, and neutrophils (arrow head) in the dermis. **D** Fourth group induced with carrageenan and orally dosed with BM-MSCs CM TiO2 NPs showing infiltration of paw dermis by a moderate number of inflammatory cells, mainly lymphocytes and eosinophils (arrow). **E** Fifth group induced with carrageenan and orally dosed with mono-doped BM-MSCs CM TiO_2_ NPs showing mild edema in paw dermis (blue arrow) with infiltration by a few mononuclear inflammatory cells (black arrow). **F** Sixth group induced with carrageenan and orally dosed with dual-doped BM-MSCs CM TiO_2_ NPs, showing normal histological structure of paw epidermis and dermis. (hematoxylin and eosin stain, X400)

Photomicrographs of paw tissue sections stained with hematoxylin and eosin, illustrating the histopathological alterations among the experimental groups in Fig. 7. The healthy control group (A) exhibited a normal histological architecture of the epidermis and dermis, with intact connective tissue and absence of inflammatory infiltrates (Cao et al. 2024). In contrast, the positive carrageenan-induced group (B) showed severe pathological alterations, including marked dermal hemorrhage (star) and dense infiltration of inflammatory cells—predominantly lymphocytes and eosinophils (arrow)confirming successful induction of acute inflammation (Zoheir et al. 2025).

The indomethacin-treated group (C) demonstrated partial protection, but still revealed areas of necrosis accompanied by heavy infiltration of mixed inflammatory cells, including lymphocytes, eosinophils, and neutrophils (arrow head), indicating that inflammation was not completely resolved at this stage. The TiO₂-treated group (D) displayed moderate infiltration of inflammatory cells within the dermis, reflecting a noticeable yet incomplete anti-inflammatory response.

A clearer improvement was observed in the mono-doped TiO₂ group (E), which showed only mild dermal edema (blue arrow) with limited infiltration by a few mononuclear inflammatory cells (black arrow), signifying significant attenuation of carrageenan-induced inflammation. Remarkably, the dual-doped TiO₂-treated group (F) exhibited nearly normal epidermal and dermal structures, with restoration of tissue integrity and absence of inflammatory infiltration, closely resembling the normal control group (Abd-Rabou et al. 2016a).

## Conclusion

This study demonstrates that carrageenan-induced inflammation leads to increased expression of pro-inflammatory markers such as IL-4, IL-6, IL-10, TNF-α, COX-2, and 5-LOP via NF-κB pathway activation, while also inducing oxidative stress by generating reactive oxygen species (ROS), which depletes antioxidant enzymes (CAT, SOD, GPx). This disruption exacerbates tissue damage and inflammation. Treatment with TiO₂ nanoparticles, particularly those doped with Cu and Zn, effectively mitigates inflammation by inhibiting the NF-κB pathway, reducing cytokine expression, and restoring antioxidant balance through Nrf2/HO-1 pathway activation, leading to enhanced cytoprotective enzyme production. The study further highlights the regenerative potential of bone marrow-mesenchymal stem cells (BM-MSCs). Their secretome, delivered via conditioned media (CM), exhibits significant anti-inflammatory and antioxidant properties, reducing inflammatory mediator levels while enhancing antioxidant enzyme activity. The use of BM-MSC-CM offers an effective, cell-free therapeutic approach with lower immunogenicity and greater ease of application. Among the tested treatments, dual-doped TiO₂ nanoparticles (Cu and Zn) exhibited the strongest anti-inflammatory and antioxidant effects, followed by mono-doped TiO₂ (Cu), with TiO₂ alone being the least effective. All treatments showed significant reductions in inflammatory and oxidative markers, with effects comparable to the standard anti-inflammatory drug, indomethacin. These findings underscore the therapeutic potential of modified TiO₂ nanoparticles and BM-MSC-derived secretome as promising strategies for managing inflammation and oxidative stress.

## Limitations of study

The study has several limitations that may impact its clinical translation and broader applicability. The use of Sprague Dawley rats as an animal model may not fully replicate human inflammatory and oxidative responses. Additionally, the study is short-term, assessing only single-dose effects without evaluating long-term efficacy, safety, or potential toxicity. While cytotoxicity is measured using the MTT assay, this method does not assess apoptosis, genotoxicity, or prolonged cellular effects. The proposed mechanisms involving the NF-κB and Nrf2/HO-1 pathways lack direct molecular validation through techniques such as Western blotting or gene silencing. Variability in conditioned media (CM) composition due to culture conditions may lead to inconsistencies in therapeutic effects. The study relies solely on carrageenan-induced inflammation, limiting the generalizability of results, and uses only indomethacin as a reference drug, without comparing TiO_2_ treatments to other anti-inflammatory agents. Furthermore, the absence of pharmacokinetic and biodistribution studies leaves uncertainties regarding the absorption, metabolism, and systemic effects of TiO_2_ nanoparticles in vivo*.*

## Data Availability

The data that support the findings of this study are available from the corresponding author upon reasonable request.

## Abbreviations

5-LOP: Lipoxygenase inhibition activity
AP-1: Activating protein-1
BMMSCs: Bone marrow mesenchymal stem cells
CAT: Catalase
CM: Conditioned media
CT: Control group
DAMPs: Damage-associated molecular patterns
DMEM: Dulbecco’s modified eagle medium
DMSO: Dimethyl sulfoxide
ELISA: Enzyme-linked immunosorbent assay
FBS: Fetal bovine serum
FRAP: Ferric reduction antioxidant power
GPx: Glutathione peroxidase
GSH: Glutathione
GSSG: Oxidized glutathione
HO-1: Heme oxygenase 1
IL-1β: Interlukin-1β
IRAK: IL-1 receptor-associated kinase
Keap 1: Kelch-like ECH-associated protein 1
LD: Lethal dose
LPS: Lipopolysaccharide
MDA: Malondialdehyde
MSCs: Mesenchymal stem cells
MTT: 3-(4, 5-Dimethylthiazol-2-yl)-2, 5-diphenyltetrazolium bromide
MYD88: Myeloid differentiation primary response protein 88
NF-κB: Nuclear factor kappa B
NO: Nitric oxide
Nrf 2: Nuclear factor erythroid 2-related Factor 2
PAMPs: Pathogen-associated molecular patterns
PI: Propidium iodide
SD: Standard deviation
SH: Sulfhydryl
SOD: Superoxide dismutase
TAC: Total antioxidant capacity
TEM: Transmission electron microscopy
TiO_2_: Titanium dioxide
TLR: Toll like receptor
TLR4/NF-κB: Toll-Like receptor 4/nuclear factor kappa B
TNF-α: Tumor necrosis factor- α
XRD: X-ray diffraction
